# Unveiling the immunological functions of the RNA m^5^C reader YBX1 in cancer

**DOI:** 10.3389/fimmu.2025.1703697

**Published:** 2025-11-19

**Authors:** Chunhong Li, Yixiao Yuan, Xiulin Jiang, Qiang Wang

**Affiliations:** 1 Department of Oncology, Suining Central Hospital, Suining, Sichuan, China; 2 Department of Systems Biology, City of Hope Comprehensive Cancer Center Biomedical Research Center, Monrovia, CA, United States; 3 Department of Gastrointestinal Surgical Unit, Suining Central Hospital, Suining, Sichuan, China

**Keywords:** YBX1, cancer progression, tumor immune regulation, tumor microenvironment, therapeutic target

## Abstract

Y-box binding protein 1 (YBX1) is a multifunctional RNA-binding protein that plays a central role in cancer progression across diverse tumor types. Mechanistically, YBX1 promotes tumorigenesis through multiple pathways, including interactions with long noncoding RNAs (lncRNAs) and circular RNAs (circRNAs) to regulate gene transcription and translation, direct protein-protein interactions to stabilize oncogenic factors or facilitate their nuclear translocation, and recognition of RNA modifications such as m^5^C to enhance mRNA stability and translation. Beyond intrinsic tumor cell functions, YBX1 also modulates the tumor immune microenvironment. It can regulate immune cell activity and the efficacy of immune checkpoint inhibitors in both m^5^C-dependent and -independent manners, thereby influencing responses to immunotherapy. Collectively, YBX1 acts as a critical driver of cancer progression and therapeutic resistance, integrating epitranscriptomic regulation, RNA-protein interactions, and immune modulation. These insights highlight YBX1 as a promising biomarker and potential target for combination therapies in cancer treatment.

## 1 Introduction

Y-box binding protein 1 (YBX1) is a highly conserved and multifunctional member of the cold-shock protein superfamily, whose unique structural features underpin its pivotal roles in diverse biological processes (1). The YBX1 protein is primarily composed of three domains: an N-terminal alanine/proline-rich region, a central cold-shock domain (CSD), and a C-terminal region enriched in acidic and basic amino acids (2). Among these, the CSD represents the core functional domain of YBX1, capable of binding both DNA and RNA, while the C-terminal domain mediates multiple protein–protein interactions, thereby conferring broad and complex regulatory functions (3). Functionally, YBX1 serves as a key regulator of gene expression, exerting control at multiple levels, including transcription, translation, and mRNA stability (4). On one hand, YBX1 can modulate the transcriptional activity of oncogenes and tumor suppressor genes by binding to their promoter regions; on the other hand, it plays critical roles in mRNA splicing, transport, and translation (5). Moreover, YBX1 can bind and stabilize mRNAs under stress conditions, promoting cell survival and adaptation, thereby playing essential roles in stress responses and disease pathogenesis (5).

In recent years, accumulating evidence has highlighted the central role of YBX1 in cancer development and progression. YBX1 not only contributes to tumor cell proliferation, invasion, and metastasis but is also closely associated with resistance to radiotherapy, chemotherapy, and targeted therapies (6). In addition, YBX1 plays a significant role in tumor immune regulation by influencing the expression of immune checkpoint molecules, modulating the tumor microenvironment (TME), and indirectly affecting the function of immune effector cells such as T cells and macrophages, thereby facilitating tumor immune evasion (7).

Emerging studies have established YBX1 as a direct reader of RNA 5-methylcytosine, integrating epitranscriptomic regulation with its RNA-binding and protein-interaction capacities. Beyond m^5^C recognition, YBX1 cooperates with other RNA-binding proteins and m^6^A readers to fine-tune transcript stability, splicing, and translation, while its phase separation and multivalent protein interactions enable context-dependent transcriptional regulation. This RNA-centered framework provides a mechanistic link between YBX1 activity and immune evasion, including modulation of the PD-L1 axis and remodeling of myeloid populations within the TME. Building on this perspective, the present review systematically summarizes the multifaceted roles of YBX1 in tumor biology and immune regulation, highlighting its potential as a therapeutic target and the challenges in translating these insights into clinical applications.

## 2 DNA and RNA binding functions of YBX1

**Figure 1:**
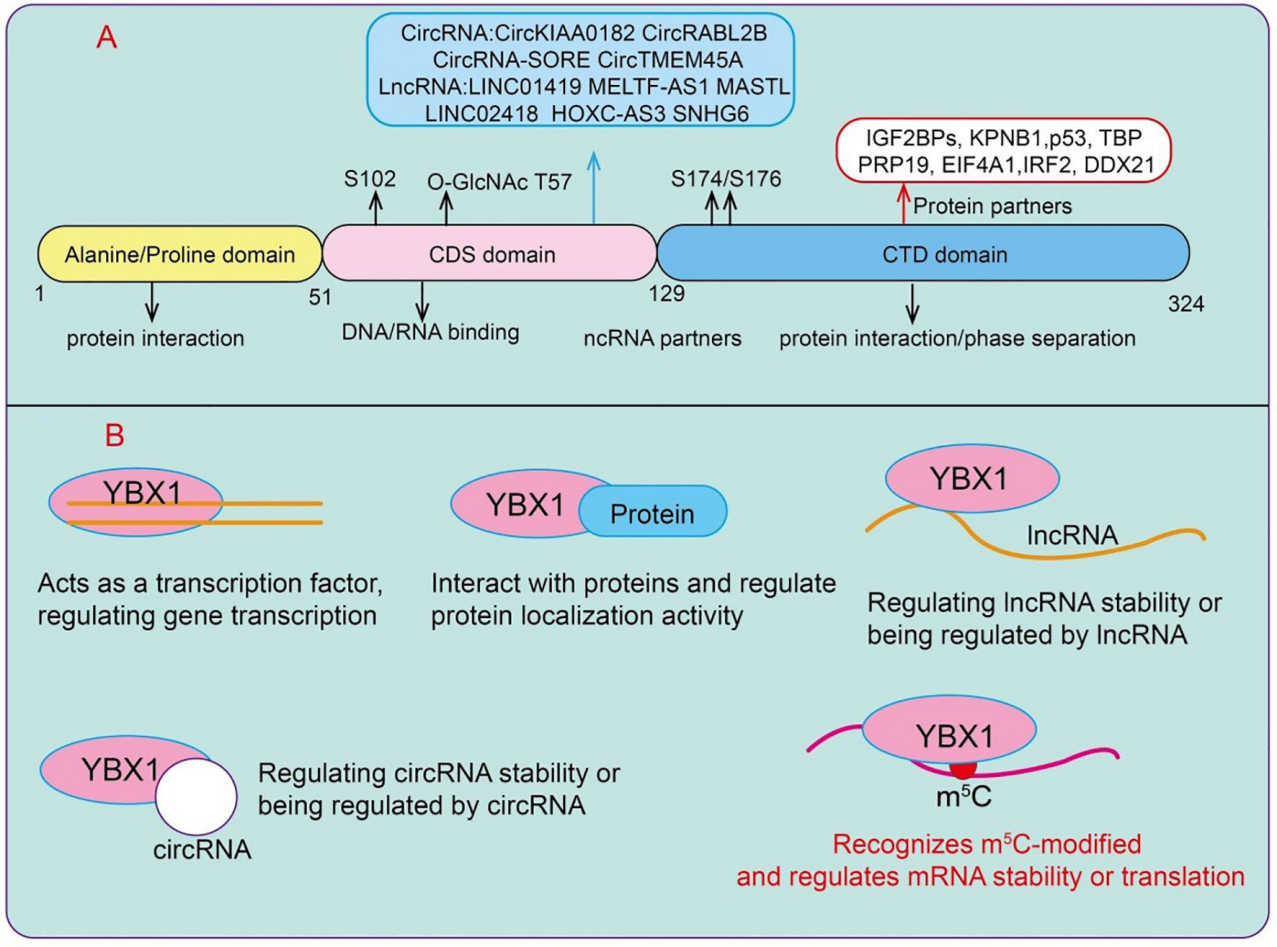
Functions and mechanisms of YBX1. **(A)** The domain architecture of YBX1. **(B)** The molecular mechanisms underlying its functions include interactions with proteins, binding with lncRNAs and circRNAs, transcriptional regulation, and its role as a reader of RNA m^5^C modifications.

Y-box binding protein 1 (YBX1) is a 36 kDa protein composed of three domains: an N-terminal alanine/proline-rich domain (A/P domain), a central cold-shock domain (CSD), and a large C-terminal domain (CTD), which alternate in positively and negatively charged amino acid residues (8) (**Figure 1A**). As a dual nucleic acid-binding protein, YBX1 can interact with both DNA and RNA, a feature that enables it to play critical roles at multiple regulatory layers, including transcriptional control, mRNA processing, and stability maintenance (8). YBX1 is a multifunctional protein whose name derives from its ability to bind to the Y-box DNA sequence present in the promoter regions of numerous genes, thereby directly or indirectly regulating their expression (9). Although initially recognized as a DNA transcription factor, YBX1 has over the years been implicated in a wide array of functions, ranging from DNA repair to precursor mRNA splicing, translation, and packaging (10). YBX1 predominantly localizes in the cytoplasm, but upon exposure to ultraviolet radiation or certain chemotherapeutic agents such as cisplatin, it translocates to the nucleus.

YBX1 functions as a bona fide reader of RNA m^5^C, selectively recognizing and binding to m^5^C-modified transcripts through its cold-shock domain (CSD). Structural and biochemical studies suggest that the CSD adopts a β-barrel fold that accommodates the methylated cytosine, enabling sequence- and modification-specific interactions that enhance transcript stability and translation efficiency. By directly binding m^5^C -modified RNAs, YBX1 protects these transcripts from degradation, facilitates their cytoplasmic localization, and promotes their efficient translation, thereby linking RNA epitranscriptomic marks to post-transcriptional gene regulation (11, 12). Although YBX1 does not directly recognize N6-methyladenosine (m^6^A), it plays a critical role in m^6^A-dependent regulation through cooperative interactions with m^6^A reader proteins, particularly members of the IGF2BP family (13). In this context, YBX1 forms ribonucleoprotein complexes with IGF2BPs on m^6^A -modified transcripts, stabilizing key oncogenic mRNAs such as MYC and BCL2 in acute myeloid leukemia (AML) (13). This functional synergy allows YBX1 to indirectly modulate the fate of m^6^A -marked transcripts, extending its influence across multiple RNA epitranscriptomic layers (13). It can regulate downstream gene expression through interactions with long non-coding RNAs (lncRNAs) or circular RNAs (circRNAs), and it can participate in post-transcriptional regulation by interacting with protein complexes or acting as a reader of m^5^C RNA modifications (**Figure 1B**).

## 3 Role and mechanisms of YBX1 in tumor immune regulation

### 3.1 Regulation of immune cell function

#### 3.1.1 Suppression of CD8^+^ T cell activation and cytotoxicity

**Figure 2:**
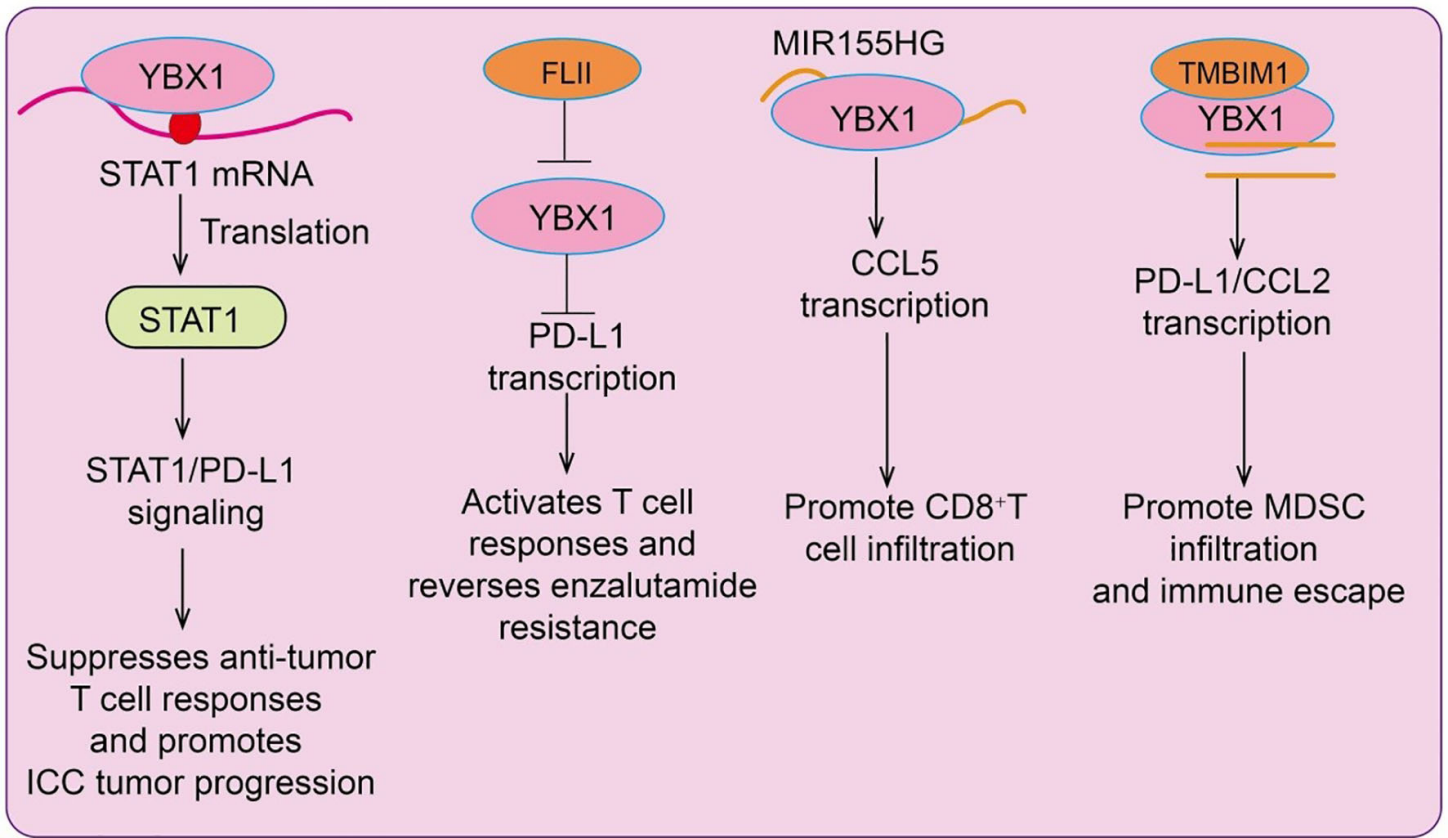
Schematic illustration of YBX1-mediated transcriptional and post-transcriptional regulation of immune responses in cancer. YBX1 promotes tumor immune modulation through multiple mechanisms. (Left) YBX1 enhances STAT1 mRNA translation, activating STAT1/PD-L1 signaling to suppress anti-tumor T cell responses and promote intrahepatic cholangiocarcinoma (ICC) progression. (Middle left) The YBX1–FLII complex upregulates PD-L1 transcription, activating T cell responses and reversing enzalutamide resistance. (Middle right) YBX1 binds to MIR155HG to induce CCL5 transcription, promoting CD8^+^ T cell infiltration. (Right) Interaction between YBX1 and TMBIM1 enhances PD-L1 and CCL2 transcription, facilitating MDSC infiltration and tumor immune escape.

Intrahepatic cholangiocarcinoma (ICC), the second most common primary liver cancer after hepatocellular carcinoma (HCC), exhibits high YBX1 expression, which correlates with poor patient prognosis. Mechanistically, YBX1 acts as an m^5^C (5-methylcytosine) RNA reader to enhance STAT1 translation, thereby activating the STAT1/PD-L1 pathway (14). This suppresses anti-tumor T cell responses and promotes tumor progression, suggesting that YBX1 facilitates immune evasion in ICC via regulation of RNA modifications and immune checkpoint signaling, providing a potential target for immunotherapy. In prostate cancer, endocrine therapy-resistant tumor cells evade immune surveillance and cytotoxicity. Downregulation of Flightless I homolog (FLII) during resistance leads to activation of the YBX1/PD-L1 axis (15), generating an immunosuppressive tumor microenvironment. FLII expression inversely correlates with PD-L1 levels in tumors. Mechanistically, FLII physically interacts with YBX1 to inhibit its nuclear localization, suppressing PD-L1 transcription. Restoration of FLII expression in resistant tumors reactivates CD8^+^ T cell responses, reverses enzalutamide resistance, enhances effector T cell proliferation, and reduces regulatory T cell and myeloid-derived suppressor cell (MDSC) infiltration (15). MIR155HG, an oncogenic lncRNA in multiple cancers, is highly expressed in lung adenocarcinoma (LUAD) and positively correlates with patient overall survival. MIR155HG interacts with YBX1 to enhance CCL5 transcription and stabilizes YBX1 by inhibiting its ubiquitination, thereby promoting CD8^+^ T cell infiltration (16). Moreover, MIR155HG upregulates PD-L1 to suppress the activity of recruited CD8^+^ T cells; this inhibitory effect can be reversed by anti-PD-L1 therapy. Clinically, patients with high MIR155HG expression show improved response to immunotherapy, and combined overexpression of MIR155HG with PD-L1 blockade enhances therapeutic efficacy (16). Chemotherapy-resistant tumor cells often escape immune-mediated destruction. YBX1 is upregulated in chemoresistant tumors and contributes to multidrug resistance while promoting an immunosuppressive microenvironment via PD-L1 upregulation. In HCC, high YBX1 expression correlates negatively with overall survival and positively with PD-L1 expression. Mechanistically, YBX1 binds specific sequences on the PD-L1 promoter, directly activating its transcription (17). YBX1 knockdown inhibits PD-L1 expression, restores functional cytotoxic CD8^+^ T cells, reduces MDSC and regulatory T cell infiltration, reverses chemoresistance, and reinstates anti-tumor immunity (17). These findings highlight YBX1 as a dual regulator of immune evasion and drug resistance, offering a potential therapeutic target in chemoresistant cancers. Interestingly, in non-tumor contexts such as atrial fibrillation (AF) and sleep deprivation (SD), machine learning approaches identified YBX1 as a key diagnostic gene. Immune infiltration analyses revealed strong correlations between YBX1 expression and specific immune cells, particularly CD8^+^ T cells and M1 macrophages (18). *In vivo* studies demonstrated that sleep deprivation exacerbates atrial remodeling and inflammatory cell infiltration, accompanied by increased YBX1 and inflammasome components, indicating a key role of YBX1 in immune regulation and inflammatory responses(**Figure 2**).

#### 3.1.2 Polarization of tumor-associated macrophages

Immunotherapy in pancreatic cancer faces challenges due to the highly immunosuppressive tumor microenvironment. GFPT2, a key enzyme in the hexosamine biosynthesis pathway (HBP), acts as an immune-related prognostic gene by promoting M2 macrophage polarization and enhancing tumor malignancy. HBP generates UDP-GlcNAc for protein O-GlcNAcylation, and GFPT2-mediated O-GlcNAcylation plays a critical role in regulating the immune microenvironment (19). Mechanistically, YBX1 undergoes nuclear translocation via GFPT2-mediated O-GlcNAcylation and functions as a transcription factor to promote IL-18 transcription, thereby modulating the immune milieu.

#### 3.1.3 Myeloid-derived suppressor cells

Pancreatic ductal adenocarcinoma (PDAC) is characterized by a profoundly immunosuppressive tumor immune microenvironment (TIME). Transmembrane protein TMBIM1 is markedly upregulated in PDAC tissues and cells, promoting tumor proliferation, migration, and growth while inducing substantial MDSC infiltration (20). Mechanistically, TMBIM1 interacts with transcription factor YBX1, enhancing YBX1 binding to the PD-L1 and CCL2 promoters, thereby upregulating their transcription. This promotes MDSC infiltration and immune evasion. Mouse models demonstrate that TMBIM1 knockdown combined with anti-PD-1 therapy achieves superior anti-tumor effects compared with anti-PD-1 monotherapy (20).

### 3.2 Regulation of immune checkpoint molecules and immunotherapy by YBX1

In lung cancer, tumor cells often evade radiotherapy through PD-L1-mediated immune escape, contributing to therapy resistance. Cyclin-dependent kinase-like 1 (CDKL1) can suppress lung cancer cell growth and proliferation while enhancing radiosensitivity (21). Mechanistically, CDKL1 interacts with the transcription factor YBX1, reducing YBX1 binding to the PD-L1 promoter, thereby inhibiting PD-L1 expression, activating CD8^+^ T cells, and preventing tumor immune escape (21). These findings highlight the critical role of YBX1 in transcriptional regulation of PD-L1 and suggest that targeting the YBX1/PD-L1 axis may enhance combined radiotherapy and immunotherapy efficacy. Pancreatic ductal adenocarcinoma (PDAC) is characterized by a highly immunosuppressive tumor immune microenvironment (TIME) with complex interactions between immune cells and heterogeneous tumor cells. Transmembrane BAX inhibitor motif-containing protein 1 (TMBIM1) is highly expressed in PDAC tissues and cell lines, promoting tumor cell proliferation, growth, and migration while inducing substantial MDSC infiltration (20). Mechanistically, TMBIM1 binds to YBX1, enhancing YBX1’s binding to PD-L1 and CCL2 promoters, thereby upregulating their transcription. This promotes MDSC infiltration and reinforces immunosuppressive TIME. Functional studies demonstrate that TMBIM1 knockdown combined with anti-PD-1 therapy achieves superior anti-tumor effects compared with monotherapy. These results indicate that the TMBIM1/YBX1 axis is a key driver of immune escape in PDAC and a potential target to improve immunotherapy responsiveness (20). Diffuse large B-cell lymphoma (DLBCL), an aggressive non-Hodgkin lymphoma, exhibits complex tumorigenesis mechanisms. NSUN2 is highly expressed in DLBCL tissues and cells. Exosomes derived from DLBCL cells transfer NSUN2 to other tumor cells, promoting proliferatio (22), M2 macrophage polarization, immune evasion, and inhibiting apoptosis. Mechanistically, NSUN2 stabilizes PD-L1 mRNA via m^5^C -dependent and YBX1-dependent pathways, enhancing PD-L1 expression. Inhibition of PD-L1 significantly attenuates the effects of exosomal NSUN2 on DLBCL proliferation, apoptosis, M2 macrophage polarization, and immune evasion (22). These findings suggest that exosomal NSUN2 promotes tumor growth through YBX1-mediated PD-L1 stabilization, highlighting YBX1 as a key immune regulator in DLBCL progression. Tumor immune escape relies on complex interactions between tumor cells and adaptive and innate immune cells, with immune checkpoints playing a central role. Long non-coding RNA TUG1 is highly expressed in hepatocellular carcinoma (HCC), driven by METTL3-mediated m^6^A modification. TUG1 knockdown suppresses tumor growth and metastasis, increases CD8^+^ T cell and M1 macrophage infiltration, enhances CD8^+^ T cell activation via PD-L1, and promotes macrophage phagocytosis through CD47 (18). Mechanistically, TUG1 acts as a miRNA sponge for miR-141 and miR-340 to regulate PD-L1 and CD47 expression and interacts with YBX1 to transcriptionally upregulate both immune checkpoint molecules, thereby modulating tumor immune escape. Clinically, TUG1 expression correlates positively with PD-L1 and CD47. Combination therapy using TUG1-siRNA and anti-PD-L1 antibody significantly suppresses tumor growth (18). IFN-γ treatment strongly induces IRGM expression in HCC, which correlates with poor patient prognosis. Functional studies show that IRGM promotes HCC malignancy. Single-cell sequencing reveals that IRGM inhibition enhances CD8^+^ cytotoxic T lymphocyte (CTL) infiltration and significantly reduces PD-L1 expression. Mechanistically, IRGM interacts with YBX1, promoting its association with phosphorylated kinase S6K1, enhancing YBX1 phosphorylation and nuclear translocation, and thereby activating PD-L1 transcription (17). Functional studies further demonstrate that IRGM inhibition combined with anti-PD-1 therapy shows superior anti-tumor efficacy compared with monotherapy. These results indicate that the IRGM/YBX1 axis suppresses CD8^+^ CTL infiltration and function via PD-L1 regulation, promoting HCC progression, and suggest a potential combination strategy with immune checkpoint blockade (ICB) (17).

The transcription factor MNX1 can increase PD-L1 expression by stabilizing its mRNA rather than activating transcription. MNX1 predominantly resides in the cytoplasm and interacts with YBX (23), enhancing YBX1 binding to PD-L1 mRNA. MNX1 knockout activates cytotoxic T cell-mediated anti-tumor immunity and sensitizes tumors to CTLA-4 blockade (23). Additionally, MNX1 promotes tumor progression via immune-independent pathways, and its expression can be upregulated by the neighboring lncRNA MNX1-AS1 through E3 ubiquitin ligase HERC2 (24). Glioblastoma (GBM) is characterized by an immunosuppressive microenvironment, yet the mechanisms underlying tumor tolerance to CD8^+^ T cell-mediated killing remain incompletely understood. Chk2 forms a CHK2-YBX1/YBX3 complex that suppresses pro-inflammatory gene transcription, promoting CD8^+^ T cell tolerance. Immunoprecipitation combined with mass spectrometry and phosphoproteomics reveals interactions between Chk2 and YBX1/YBX3, as well as positive cross-regulation in multiple glioma cell lines. Targeting YBX1 with the inhibitor SU056 degrades the CHK2-YBX1/YBX3 complex, enhancing antigen presentation and antigen-specific CD8^+^ T cell proliferation (24). Combining SU056 with ICB significantly improves survival in multiple glioma models. These results reveal a CHK2-YBX1/YBX3 complex-mediated immune suppression mechanism, suggesting that targeting YBX1 and its complexes in GBM may have clinical potential in combination with ICB therapy (24).

## 4 YBX1 as a potential therapeutic target

YBX1 plays a pivotal role in various cancers and immune-related diseases, contributing to tumor proliferation, metastasis, drug resistance, and immune evasion, making it a highly promising therapeutic target. Intervention strategies targeting YBX1 primarily include small-molecule inhibitors that block its expression or nuclear translocation, nucleic acid-based approaches, and combination therapies. Small molecules can inhibit YBX1’s nuclear translocation or interfere with its DNA/RNA binding, thereby suppressing downstream oncogenic signaling. For instance, in glioblastoma, the YBX1 inhibitor SU056 disrupts the CHK2-YBX1/YBX3 complex, enhances antigen presentation and CD8^+^ T cell activity, and, when combined with immune checkpoint blockade (ICB), significantly improves survival in preclinical models, demonstrating the feasibility and potential efficacy of small-molecule targeting of YBX1 (25).

Nucleic acid-based strategies, including siRNA, antisense oligonucleotides (ASO), and proteolysis-targeting chimeras (PROTACs), offer versatile tools for YBX1 targeting. siRNA and ASO specifically degrade YBX1 mRNA to reduce protein expression, while PROTACs induce ubiquitin-mediated degradation of YBX1, enabling rapid and efficient protein clearance, thereby reversing tumor drug resistance and immune suppression (26). These approaches have shown robust anti-tumor activity and immunomodulatory potential in both *in vitro* and *in vivo* studies. Furthermore, combination therapies targeting YBX1 provide additional therapeutic opportunities. In immunotherapy, YBX1 inhibition reduces PD-L1 expression and alleviates tumor immunosuppression, thereby enhancing the efficacy of ICIs. In metabolic interventions, YBX1-mediated hexosamine biosynthesis pathway (HBP) reprogramming in pancreatic ductal adenocarcinoma can be countered with metabolic inhibitors to improve immune responses. Additionally, YBX1 inhibition can reverse chemotherapy or targeted therapy resistance, increasing drug sensitivity and achieving multi-layered anti-tumor effects.

In summary, as a key transcription factor and RNA-binding protein, YBX1 not only plays a central role in tumor progression and immune evasion but also represents a promising therapeutic target. Small-molecule inhibitors, nucleic acid-based strategies, and combination regimens collectively provide novel avenues for treating refractory tumors and immune-related diseases, highlighting its translational potential in clinical oncology.

## 5 Advantages and challenges of targeting YBX1 in cancer therapy

As a multifunctional transcription factor and RNA-binding protein, YBX1 plays a central role in tumor initiation, progression, drug resistance, and immune evasion across various cancer types, making it a promising therapeutic target. Targeting YBX1 offers several distinct advantages. First, it can simultaneously modulate multiple oncogenic pathways, including tumor proliferation, migration, metastasis, chemotherapy resistance, and the immunosuppressive microenvironment, thereby achieving multidimensional anti-tumor effects. For instance, YBX1 inhibition can reduce PD-L1 expression, remodel the immunosuppressive tumor microenvironment, and enhance the efficacy of immune checkpoint inhibitors (ICIs), while also reversing chemoresistance and increasing tumor sensitivity to anticancer drugs (18). Additionally, YBX1 is frequently overexpressed in diverse cancers and correlates with poor prognosis, making it a clinically detectable biomarker and an ideal candidate for precision-targeted therapies. However, clinical application of YBX1-targeted strategies faces several challenges. First, YBX1 is structurally multifunctional and widely distributed in both the nucleus and cytoplasm, making direct inhibition or blockade of nuclear translocation technically challenging. Second, YBX1 participates in various physiological processes, and global inhibition may cause potential side effects, necessitating highly specific and controllable intervention approaches. Third, current therapeutic modalities targeting YBX1, including small-molecule inhibitors, nucleic acid-based interventions (e.g., siRNA, ASO), and PROTACs, are still in early developmental stages, with pharmacokinetics, *in vivo* stability, and clinical safety requiring thorough evaluation. Furthermore, tumor heterogeneity and compensatory signaling pathway activation may attenuate the efficacy of YBX1-targeted therapies, indicating that single-target approaches may be insufficient to fully block its oncogenic functions (27).

In summary, targeting YBX1 in cancer therapy offers multiple advantages, including multidimensional inhibition of tumor progression, remodeling of the immune microenvironment, and reversal of drug resistance. Nevertheless, challenges such as target specificity, potential adverse effects, and tumor heterogeneity must be addressed. Future optimization of small-molecule inhibitors, nucleic acid-based approaches, and combination therapies holds promise for fully exploiting YBX1 as a precision therapeutic target in oncology.
